# Overcoming the Energy vs Power Dilemma in Commercial Li-Ion Batteries via Sparse Channel Engineering

**DOI:** 10.1021/acsenergylett.4c01727

**Published:** 2024-09-24

**Authors:** Doyoub Kim, Alexandre Magasinski, Yueyi Sun, Baolin Wang, Aashray Narla, Seung-Hun Lee, Hana Yoo, Samik Jhulki, Ah-Young Song, Jinho Hah, Ting Zhu, Alexander Alexeev, Gleb Yushin

**Affiliations:** † School of Materials Science and Engineering, Georgia Institute of Technology, Atlanta, Georgia 30332, United States; ‡ Woodruff School of Mechanical Engineering, Georgia Institute of Technology, Atlanta, Georgia 30332, United States; § SDI R&D Center, Samsung SDI, 130, Samsung-ro, Yeongtong-gu, Suwon-si, Gyeonggi-do 16678, Republic of Korea

## Abstract

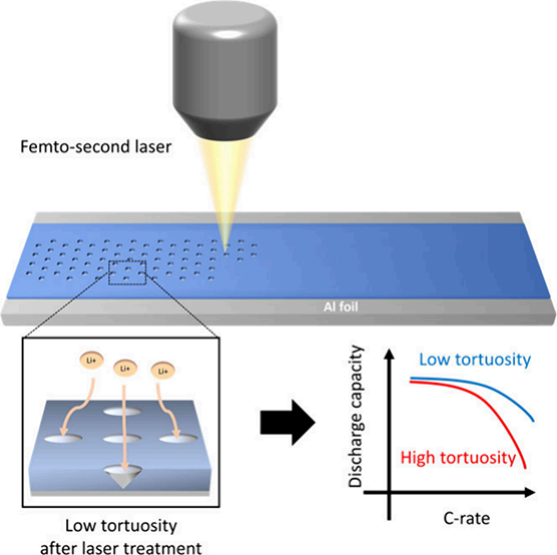

Improvements in both the power and energy density of lithium-ion batteries (LIBs) will enable longer driving distances and shorter charging times for electric vehicles (EVs). The use of thicker and denser electrodes reduces LIB manufacturing costs and increases energy density characteristics at the expense of much slower Li-ion diffusion, higher ionic resistance, reduced charging rate, and lower stability. Contrary to common intuition, we unexpectedly discovered that removing a tiny amount of material (<0.4 vol %) from the commercial electrodes in the form of sparsely patterned conical pores greatly improves LIB rate performance. Our research revealed that upon commercial production of high areal capacity electrodes, a very dense layer forms on the electrode surface, which serves as a bottleneck for Li-ion transport. The formation of sparse conical pore channels overcomes such a limitation, and the facilitated ion transport delivers much higher power without reduction in the practically attainable energy. Diffusion and finite element method-based simulations provide deep insights into the fundamentals of ion transport in such electrode designs and corroborate the experimental findings. The reported insights provide a major thrust to redesigning automotive LIB electrodes to produce cheaper, longer driving range EVs that retain fast charging capability.

To meet the growing demand for high energy density and power density in Li-ion batteries (LIBs) for electric vehicle (EV) applications (particularly in EVs offering a long driving range of 400–700 miles), production of lower cost, higher energy density cells is critically needed. The use of thick (80–100 μm) and dense electrodes with high areal capacity (≥6 mAh/cm^2^) is considered a promising solution to increase cell energy and simultaneously reduce production costs.^1−3^ This is because such designs reduce the volume and mass fractions of many inactive components (separators, aluminum (Al) and copper (Cu) current collectors, etc.). The EV battery cost reductions come both directly from savings on such materials in cells and indirectly from increased cell volumetric and gravimetric energy densities and thus reduced number of cells and a smaller and cheaper battery (including a smaller and cheaper battery safety/management system) needed in an EV to attain the same range.^4−6^

The highest performance cathodes currently in use or under the final stages of development for EV LIBs include nickel (Ni)-rich lithium nickel manganese cobalt oxides (NMC, such as NMC811 or LiNi_0.8_Mn_0.1_Co_0.1_O_2_ with practical capacity of up to ∼190 mAh/g) and Ni-rich lithium nickel cobalt aluminum oxides (NCA, such as LiNi_0.8_Co_0.15_Al_0.05_O_2_ with practical capacity of up to ∼200 mAh/g).^7−10^ To maximize the volumetric cathode capacity, such cathodes are typically densified to retain <20% porosity. When such electrodes are produced with high areal capacity loadings, their rate performance become sluggish due to the ion diffusion time being proportional to the square of the diffusion path, which is proportional to the electrode thickness and the tortuosity.^11^ If the tortuosity were thickness-independent, then an increase in cathode areal loading by 40% would increase diffusion time by ∼2×, thus dramatically reducing rate performance during both charge and discharge. This, in turn, may also jeopardize LIB cycle life or safety because slow ion transport may induce uneven delithiation of the cathode or (in case of similarly slow ion transport within the anode) nonuniform lithiation and localized Li plating on the anode, particularly at high current densities or at lower temperatures.^12^ In addition, the LIB energy and electrode capacity harvested at fast rates would also be limited, thus reducing practically attainable EV range. If the tortuosity increases with cathode thickness, such undesirable effects would be further enhanced.

Introduction of straight pore channels into the cathode can potentially increase C-rate performance.^13,14^ Yet, only if the gains in accessible capacity due to a faster ion transport could be made substantially higher than the loss of volumetric capacity due to the formation of such pore channels, such efforts may be justified. Previous research demonstrated the positive impact on rate enabled by the use of laser sources to introduce large volume fractions of patterned channels within NMC electrodes.^15^ However, such work produced highly porous (up to 50%) cathodes, which were not commercially viable due to their low volumetric capacity and energy density coupled with significant losses of the very expensive active material during laser micromachining.^16−18^ As such, there remains an open question whether the formation of straight pore channels occupying much smaller volume fractions (e.g., 1–5 vol % or less) may be suitable for meaningful improvements in the cathode rate performance.

Herein, we report on a first systematic study to elucidate the impacts of laser-patterned channels of different thicknesses, widths, and spacing on achievable capacities at different C-rates in cells comprising dense and thick NCA cathodes with areal capacity up to ∼6 mAh/cm^2^. We unambiguously demonstrate that this approach may significantly improve both power density and accessible energy density in EV LIB cells.

Figure 1a shows a schematic of the laser-patterning process, where a regular array of conically shaped pore channels is introduced into an electrode. This process is rather fast; in industrial settings, it can be done much faster, with roll-to-roll before or after calendering using an array of lasers, an array of optical fibers^19^ or fiber Bragg gratings^20^ carrying the laser signal from a single more powerful source. An important advantage of this laser patterning method is clearly demonstrated in Figure 1b, where the energy density vs C-rate performance for pristine and laser-patterned high nickel NCA cathodes with 50 μm channel diameter and 100 μm channel spacing is plotted. Better rate capacity is demonstrated by the laser-patterned cathode, which retains noticeably greater energy densities when the C-rate rises from 0.1C to 2C. It is important to note that, as Table 1 in the Supporting Information illustrates, proper pattern engineering can reduce the electrode material losses for chosen designs, resulting in very small losses (0.03–7.55 vol %).

**Figure 1:**
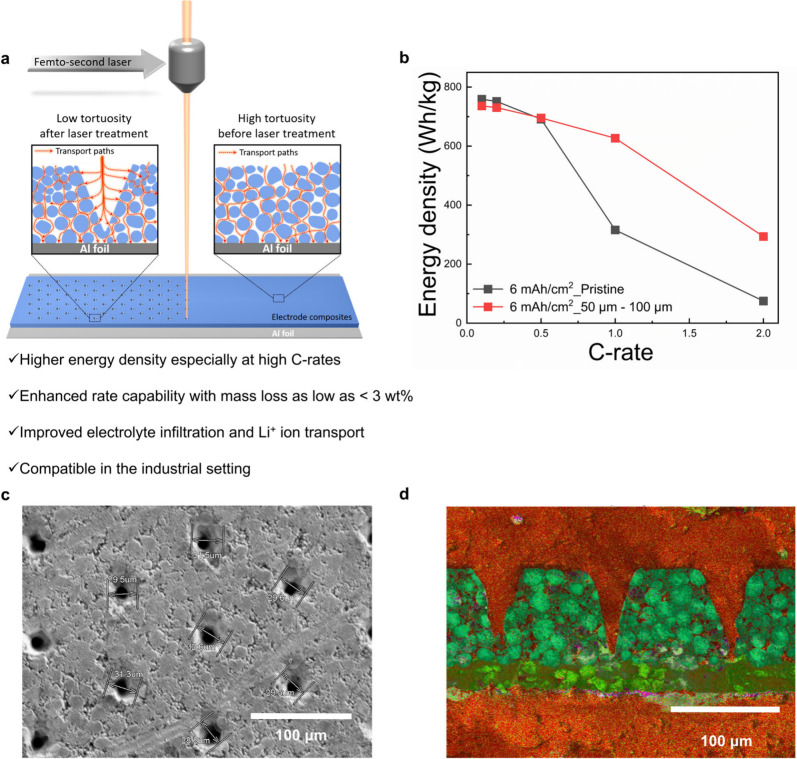
Schematic of laser patterning, its impact, and material characterization of channel formation and morphology in a 6 mAh/cm^2^ high nickel NCA electrode. (a) Schematic showing how laser treatment can lower tortuosity and improve electrolyte wetting and ion transport, thereby contributing to improvement in electrochemical rate performance of thick and dense battery electrodes. (b) Attainable energy density at high current density with a laser-patterned thick and dense electrode. The representative top-view SEM images reveal that the (c) 30 μm–100 μm hexagonal array channel patterns are successfully formed. (d) Cross-section SEM of a 30 μm–100 μm laser-patterned electrode, showing the conical-shaped holes formed. EDS mapping of the laser-patterned electrode shows the elemental mapping where red and purple represent the chemical elements that come from the epoxy preparation for cross-sectional images and light green, sky blue, dark green, and orange come from the high nickel NCA electrode and the aluminum current collector. See Figure S2 in the Supporting Information for more details on the elemental information. Note that 30 μm–100 μm refers to the top diameter of the channel and the spacing between the nearest channels, respectively.

We patterned two commercially produced thick and dense high nickel NCA cathodes (electrode density of ∼3.7 g/cm^3^, thicknesses of ∼80 and ∼95 μm and areal capacities of 4.8 and 6 mAh/cm^2^, respectively) using a fs-laser source to create channels of controllable size (width and depth) and spacing between them (see Experimental Methods in the Supporting Information). We observed a color change (darkening) of the material around the laser-cut channels induced by the residual heat from laser patterning. However, the laser patterning process does not cause any undesirable changes in the overall phase of the bulk NCA cathodes, as determined from identical powder X-ray diffraction (XRD) profiles of pristine and laser-patterned electrodes (Figure S1 in the Supporting Information) as well as Raman spectroscopy studies (Figure S3 in the Supporting Information), suggesting possible local binder carbonization.

The representative top-view scanning electron microscopy (SEM) images reveal the presence of a uniformly distributed hexagonal array of holes on the top surface (see example patterns with the surface diameter of ∼30 μm and spacing of ∼100 μm in Figure 1c). The cross-sectional optical images of the laser-patterned electrode demonstrated in Figure S3 in the Supporting Information reveal that the created channels are conical and tapered. The conical shape of the channels likely originates from the local heat distribution under the laser beam and the resulting material losses, where the electrode becomes the hottest on the top surface, while the heat is dissipated more readily near the metallic aluminum (Al) current collector. Figure 1d shows examples of such channels spaced ∼100 μm from each other and extending from the top of the cathode surface down to the Al foil. Such channels should facilitate ion transport throughout the entire electrode bulk. Cross-sectional energy-dispersive X-ray spectroscopy (EDS) mapping confirms the presence of all the expected elements (Figure S2 in the Supporting Information). From a practical standpoint, conical holes may be preferred over cylindrical holes of the same diameter due to the consideration of smaller electrode mass loss in the case of the former, while still providing an effective way for a less tortuous path for ion transport.

Electrochemical tests were conducted in half cells of 2032-type using pristine or patterned NCA cathodes, a Li metal foil anode, 1 M LiPF_6_ EC:DEC (V:V = 1:1), electrolyte and Celgard 2400 separator (see Experimental Methods in the Supporting Information) to reveal the impact of straight pore channels on the electrochemical performance, and to compare and quantify the discharge rate capabilities of the pristine and laser-patterned samples with different channel spacings and sizes. Compared to the pristine electrode, the laser-patterned cathodes resulted in noticeably improved rate performance, showing higher rate capacities at current densities in the range from 1C to 3C (Figures 2a–c) likely due to faster ion transport throughout the bulk electrode. To effectively compare the values for the electrodes with an areal capacity of 4.8 mAh/cm^2^, the discharge capacities obtained from each half cell for high C-rates were normalized by the discharge capacity obtained at a 0.1C (slow) rate (Figure 2d). The results clearly show that, beyond 0.5C, the channels begin to noticeably influence the achievable capacity, as we had hoped to attain. Furthermore, for the pore channel spaced at ∼100 μm the rate performance was particularly impressive, exceeding those from other samples by quite some margin, especially at faster C-rates.

**Figure 2:**
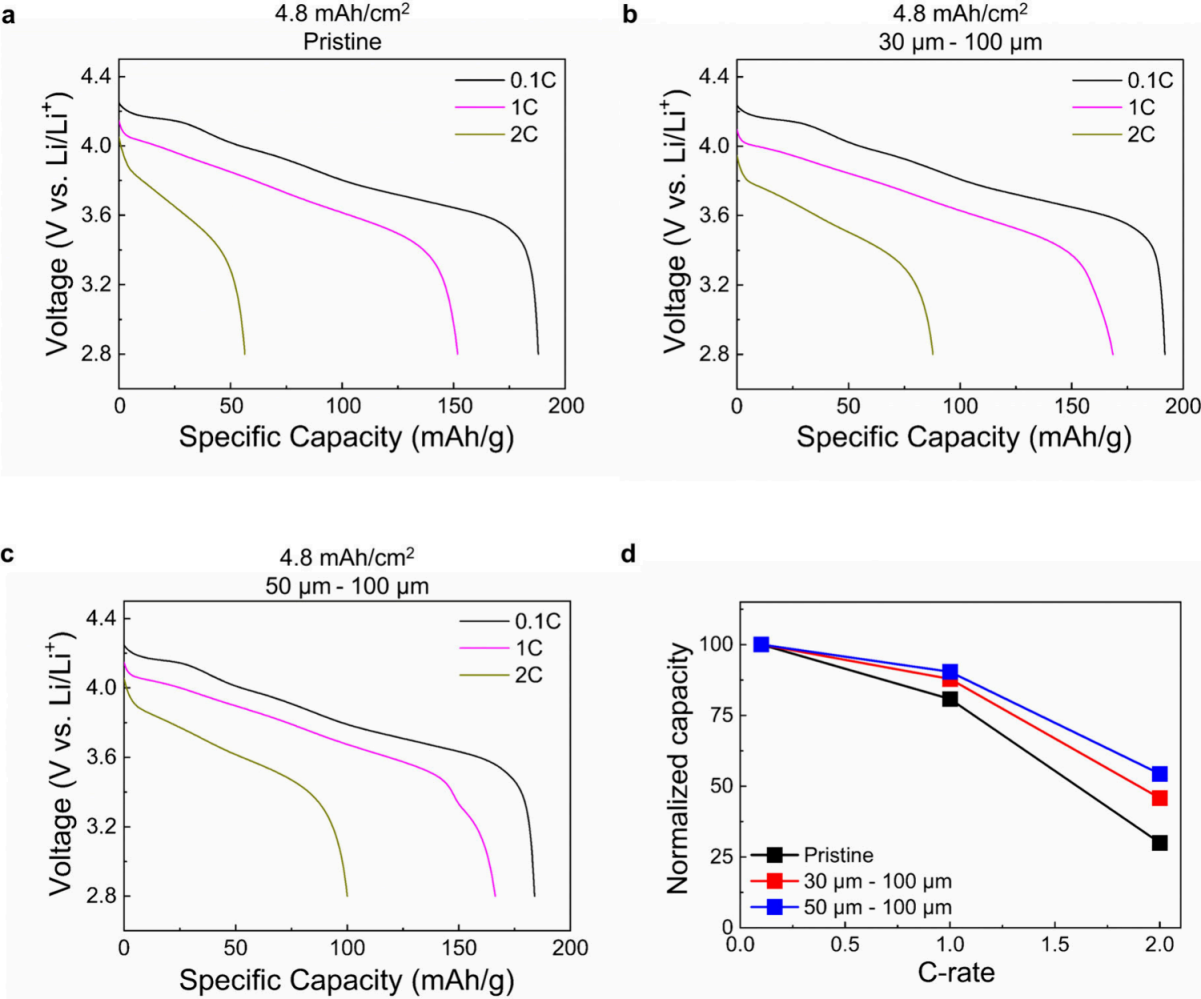
Electrochemical performance of pristine and laser-patterned 4.8 mAh/cm^2^ electrodes. Discharge rate capability tests for (a) pristine and laser-patterned (b) 30 μm–100 μm and (c) 50 μm–100 μm 4.8 mAh/cm^2^ electrodes where the charge rate was fixed at 0.1C for the 0.1C cycle and 0.2C for 1C and 2C discharge rate cycles. (d) Discharge capacities obtained in (a–c) normalized based on the capacities obtained at 0.1C.

A similar but even stronger trend was also observed with a higher capacity loading of ∼6 mAh/cm^2^ (cathode thickness ∼95 μm). For such a high loading automotive cathode, the roles of channels in the capacity improvement at high C-rates (1C and 2C) is much more evident (Figure 3). The pristine electrode (Figure 3a) exhibits limited rate capability. In contrast, the laser-patterned cathodes with 30 μm–100 μm (Figure 3b), 50 μm–100 μm (Figure 3c), and 50 μm–450 μm (Figure 3d) channel dimensions show improved rate performance, maintaining higher specific capacities at higher C-rates. It is worth noting that the 50 μm–450 μm laser-patterned electrode outperforms the pristine electrode at high C-rates even with a small material loss. Furthermore, the normalized capacity difference between pristine and 100 μm-spacing laser-patterned samples increases from approximately 2 to 4 times as the C-rate increases from 1C to 2C for the largest pores (Figure 3e,f).

**Figure 3:**
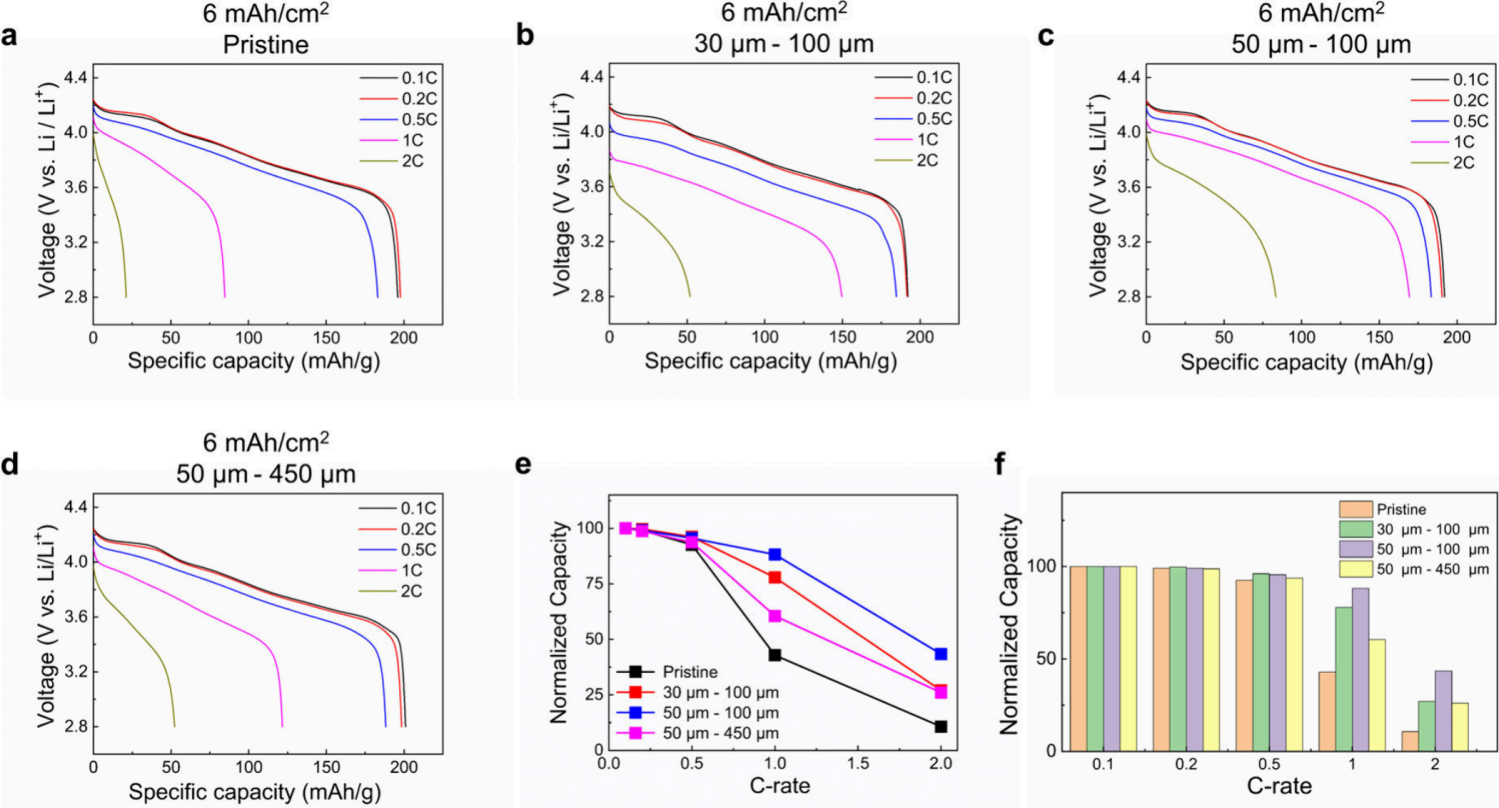
Electrochemical performance of pristine and laser-patterned 6 mAh/cm^2^ electrodes. Discharge rate capability tests for (a) pristine and laser-patterned (b) 30 μm–100 μm, (c) 50 μm–100 μm, and (d) 50 μm–450 μm 6 mAh/cm^2^ electrodes where the charge rate was fixed at 0.1C for the 0.1C cycle and 0.2C for 1C and 2C discharge rate cycles. (e) Discharge capacities obtained in (a–d) normalized by the capacities obtained at 0.1C. (f) Bar graphs of normalized capacities obtained in (e).

To further examine the roles of the channels and minimize active material loss, we fabricated an electrode sample with larger separation between the hexagonal arrays of holes (∼450 μm). Despite the spacing between the holes exceeding the electrode thickness, noticeable improvements were nonetheless obtained with such an electrode system, exhibiting higher capacities (approximately 1.45 and 2.45 times, respectively) than that of the pristine sample at 1C and 2C (Figure 3e). This indicates that the electrode tortuosity is orientation-dependent and that the ion transport is likely severely blocked at the denser or more torturous electrode top surface layer. In this case, much faster ion transport during the discharge (cathode lithiation) could be attained if the Li ions migrate throughout the electrode by initially propagating vertically through the ordered channels and then laterally through a less dense and less torturous electrode portion closer to the Al current collector foil. Indeed, our X-ray tomography measurements of the cathodes confirmed a higher density of the top surface electrode layer (note that Figure S5 in the Supporting Information may underestimate a fraction of the binder relative to the remaining pores since the active material has much higher density compared to the binder). The high resistivity of the top electrode channel also highlights the advantages of having the conical shape of the pore channels because a larger fraction of the more ionically resistive material is removed, while keeping the total pore volume small (note that the volume of a perfect straight cone is 1/3 of the volume of a cylinder with the same top diameter; the volume of the convex cone is even smaller).

Note that ion-transport pathways should be considered in both vertical and horizontal directions; while vertical ion transport is clearly much faster though the formed pore channels, the kinetics of the overall ion transport may eventually become limited by horizontal ion transport ability if the pore spacing is too large. The ideal size and space distribution of the pore channels should thus depend on the distribution of the active material and binder throughout the dense calendered electrode and the resulting distributions of pore sizes and pores shapes (that are being filled with electrolyte during cell operation). For very uniform electrodes (with the top electrode portion having equal density to the bottom electrode portion) smaller and more narrowly spaced pore channels (with the spacing smaller than electrode thickness) may be beneficial. However, for commercially produced electrodes evidently having a denser top surface layer larger spacing (larger than the electrode thickness) may still be highly advantageous. Such an electrode pattern is commonly easier and faster to make via laser-drilling. In addition, the total volume of the electrode removed by the laser may become substantially smaller (Table 1 in the Supporting Information). We hypothesize that the undesirable dense surface layer may be formed by hot roller calendering, which should make the binder near the top electrode surface hotter and thus softer and more deformable.

To elucidate the effect of laser patterning on tortuosity, MacMullin numbers—which reflect the tortuosity factor of the electrodes—were derived based on the impedance tests conducted on symmetric cells in non-Faradaic conditions (Figure 4). The laser patterning contributes to the substantial reduction in MacMullin number, when both the channel size increases and the channel spacing decreases, confirming less tortuous ion transport pathways for patterned electrodes. The 4.8 mAh/cm^2^ electrode patterned with a very large spacing of 900 μm shows a very small difference in MacMullin number, even for larger pore channels of ∼100 μm (Figure 4a, Table 2 in the Supporting Information). As the spacing decreases to 450, 250, and 100 μm, the reduction in the MacMullin number becomes more apparent (Figure 4b, Table 2 in the Supporting Information). With 50 μm conical channel electrodes, the MacMullin number decreases from ∼24.1 to ∼18.7 when the spacing decreases from 900 μm down to 100 μm, which is substantially smaller than that of the pristine NCA electrode (∼25) (Figure 4a,b, Table 2 in the Supporting Information). This trend becomes even stronger for the thicker and higher areal capacity 6 mAh/cm^2^ electrodes, (Figure 4c,d, Table 3 in the Supporting Information) demonstrating that the rate limiting ion transport resistance in thick and dense electrodes may be directly linked to the reduction in electrode tortuosity. Somewhat to our surprise, a slightly smaller MacMullin number was obtained in thicker electrodes (Figure 4), which we explained by the later generation of the coating and calendering technologies employed to produce such samples.

**Figure 4:**
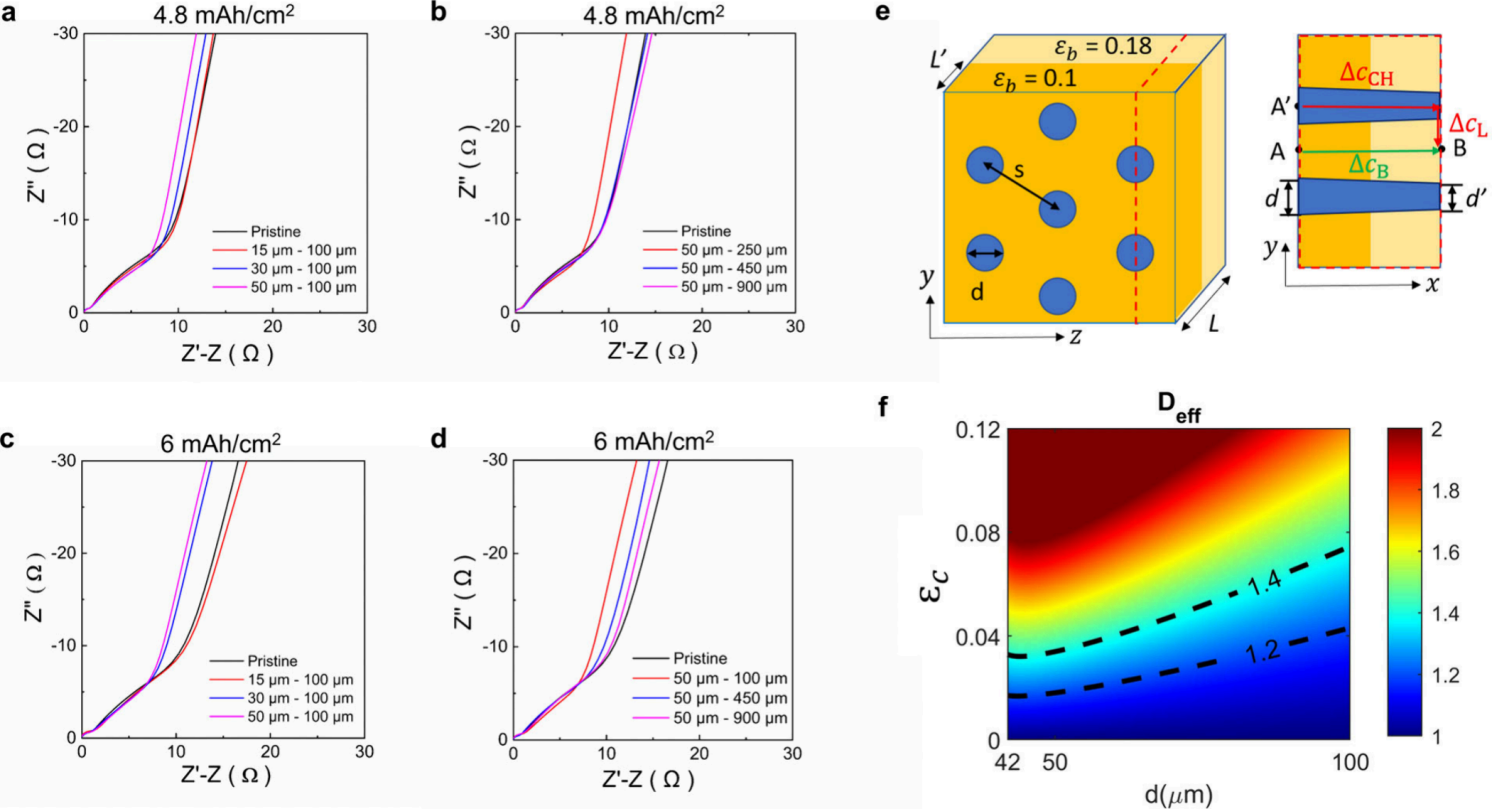
Effect of laser patterning on tortuosity and diffusivity depending on the channel diameter and spacing. The normalized Nyquist plots obtained for (a) different laser-patterned channel diameters while the channel spacing was fixed to 100 μm and for (b) different channel spacings while the channel size was fixed to 100 μm for 4.8 mAh/cm^2^ loading. The normalized Nyquist plots obtained for (c) different laser-patterned channel sizes while the channel spacing was fixed to 100 μm and (d) different channel spacing while the channel size was fixed to on 100 μm for 6 mAh/cm^2^ loading_._ Periodic array of conical channels induced by laser treatment in a porous electrode matrix. (e) Geometry of hexagonal array laser-patterned electrode with conical channels of varying porosity in the top and bottom electrode halves. The top corresponds to 10% porosity and the bottom (ε_b_) that is closer to Al foil corresponds to 18% porosity, for a total porosity of 14% for the entire electrode. (f) Contour plot of normalized effective diffusivity based on the steady-state electrolyte concentration drop across the hexagonal array laser-patterned electrode having 6 mAh/cm^2^ loading where the electrode thickness excluding the Al foil was fixed to 80 μm.

Electrode immersion tests were also conducted to compare the electrolyte impregnation ability of the laser-patterned 4.8 mAh/cm^2^ electrodes. Consistent with the trend observed for the MacMullin numbers, the laser-patterned electrodes underwent substantially faster electrolyte filling. However, the overall impact of laser patterning on the wetting rate was dramatically stronger than what we initially envisioned based on the rate performance (Figures 2 and 3) and tortuosity measurements (Figure 4). Indeed, the patterned electrodes were impregnated by a few times higher amount of electrolyte for a given amount of time (Figure 5a,b). Also note that the electrodes with smaller channel spacing show significantly faster and larger electrolyte impregnation (Figure 5b), consistent with the electrochemical performance observed for them. Taking the trends in MacMullin numbers and electrode immersion tests together, the laser-patterned electrodes with larger channel size and smaller channel spacing significantly minimize tortuosity for ionic conduction and enhance electrolyte wetting.

**Figure 5:**
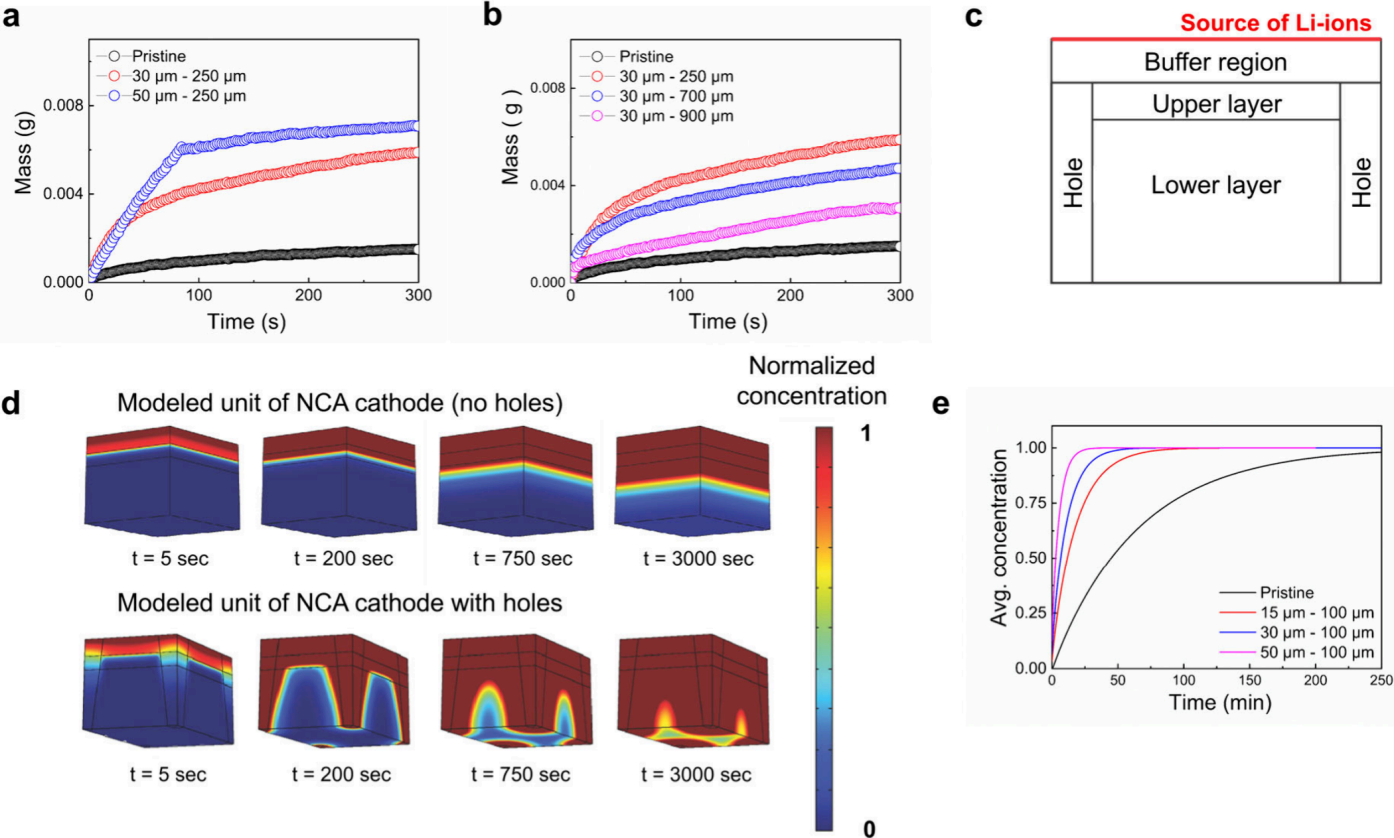
Effect of laser patterning on electrolyte wetting. Immersion tests showing electrolyte impregnation in relation to laser-patterned electrodes for variable (a) channel diameters and (b) channel spacings for the first 300 s after the electrode immersion in 1 M LiPF_6_ EC:DEC (V:V = 1:1). (c) Geometry of a single unit of NCA cathodes based on the pattern of the holes where each single unit has periodic boundaries on the sides. (d) COMSOL simulations of the pristine and laser-patterned holes significantly enhance the transportation of ions in the modeled electrode element. The unit has a side length of 100 μm and a height of 80 μm, and holes have end diameters of 20 and 40 μm. (e) Simulated results showing the time it takes for the average electrolyte concentration to equilibrate in pristine and laser-patterned electrodes.

We modeled Li-ion diffusion within a hexagonal electrode element of fixed electrode height (80 μm) and various channel diameters from 10 to 50 μm and spacings (100–700 μm) in an electrode with a denser top layer porosity of approximately 8% and a less dense layer that is closer to the Al foil with a porosity of approximately 15% to match the experimentally observed electrode in Figure S5 in the Supporting Information. The result shows that electrodes with patterned holes significantly reduce the time needed to reach a specified average Li-ion concentration as the pore channel diameter increases when the channel spacing is fixed to 100 μm (Figure 5d). The total ion count within a modeled electrode element with holes significantly exceeds those without holes until the discharging and charging time is so large that the electrodes saturate. But having too many channels with large diameters within the modeled electrode element with a small side length would reduce volumetric capacity. Removing more material leads to faster ion transportation, but one gets significantly diminishing returns after removing 8–10 vol % (Figures S7–S11).

We developed a model matrix to consider the laser-patterned electrode structure with a periodic hexagonal array of conical channels based on Figure 2d and Figure S2 in the Supporting Information, to enhance the understanding of how laser patterning with various channel sizes and spacings affects the decrease in MacMullin number. Figure 4e depicts a model structure in which the electrode thickness *L* and the porosity of the porous electrode matrix ε_b_ are fixed since they are unchanged before and after laser treatment. The remaining adjustable parameters are as follows: top hole diameter *d*, spacing *s*, and bottom hole diameter *d*′ (accounting for tapered conical channels), which collectively determine the energy density and areal capacity of the electrode. Contours for the effective diffusivity *D*_eff_ (see Experimental Methods in the Supporting Information) are shown in Figure 4f as a function of porosity resulting from the channels ε_c_ (due to the loss of active materials from laser patterning of a hexagonal array) and channel diameter *d*, for the case of a thick and dense 6 mAh/cm^2^ electrode (with a thickness of ∼80 μm excluding the Al foil). The higher the loss of electrode material, the larger the channel diameter and the smaller the channel spacing, as shown in Table 1 in the Supporting Information. It is worth noting that the black dashed lines in Figure 4f indicate that the effective diffusivity *D*_eff_ increases from 1 to 1.2, 1.4, and even higher with losses of electrode materials slightly less than 2 wt % (ε_c_ = 0.02), 4 wt % (ε_c_ = 0.04) and higher, while the channel diameter is fixed to 50 μm from laser patterning. As the electrode material loss becomes closer to 8 wt % corresponding to a channel spacing of 100 μm or less, *D*_eff_ increases from 1 to 1.6 or higher in a significant manner. This indicates that the smaller channel spacing is desirable to achieve lower tortuosity, boost electrolyte impregnation (as shown in Figure 4b), and more importantly, improve ion transport in thick and dense electrodes while tolerating some loss of electrode materials. Similarly, given that ε_c_ is slightly smaller than 0.04 in such a way that it is on the dashed line with a normalized effective diffusivity of 1.4, it would be decreased to the normalized effective diffusivity of 1.2 either by reducing electrode material loss (indicating a smaller channel spacing when the channel size is fixed to 50 μm) or by increasing the channel size while maintaining the same electrode material loss. This scenario corresponds to a design that aims to achieve the maximum rate of ion transport for a tolerable loss of active materials.

This work revealed that the formation of a dense layer on the surface of high areal capacity loading automotive cathodes significantly reduces ion transport kinetics and Li-ion battery rate performance. Such a limitation, however, could be overcome successfully by introducing sparse conical and tapered channels via laser patterning. Such channels shorten mean ion transport distance and improve electrolyte wetting. Facile vertical ion transport pathways through the straight channels followed by their horizontal transport were found to allow for much improved and homogeneous access of the ions to the electrode particles at high C-rates in thick and dense high areal loading (up to 6 mAh/cm^2^) NCA cathodes. The tradeoff between mass (volumetric capacity) loss due to laser patterning and gain in capacity retention due to fast ion transport was systematically analyzed via the creation of channels of controlled size/volume and their spacing. Even for channel holes and spacings as large as 450 μm and mass losses as little as 0.37–0.03%, significant rate improvements could be attained. This systematic study combined both experimental observation and diffusion modeling to enhance our understanding of interdependence among electrochemical performance, tortuosity, and electrolyte wetting. The insights gained will provide a major thrust to redesigning automotive Li-ion battery electrodes to attain higher power and energy density, so as to achieve cheaper, longer driving range EVs that retain fast charging capability.

## Supporting Information Available

Experimental methods, electrode material losses for selected patterns, X-ray diffraction of the electrode before and after laser patterning, EDS of the cross-section of the laser patterned electrode, Raman spectra of specific areas of a laser-patterned electrode, optical images of the cross-section of laser-patterned 6 mAh/cm^2^ electrodes, nanocomputed tomography images of the cross-sections of pristine and laser-patterned 4.8 mAh/cm^2^ electrodes, electrochemical performance of pristine and laser-patterned 4.8 and 6 mAh/cm^2^ electrodes, normalized Nyquist plots of pristine and laser-patterned 6 mAh/cm^2^ electrodes in symmetric cells, MacMullin numbers of pristine and laser-patterned 4.8 and 6 mAh/cm^2^ electrodes, experiment and simulation of electrolyte wetting and average lithium concentration, and finite element method based simulation on electrodes with difference thicknesses and uniform porosity (PDF)
