# Supplementary material for: Overcoming the Energy vs Power Dilemma in Commercial Li-Ion Batteries via Sparse Channel Engineering
Source: ACS Energy Lett. 2024 Sep 24;9(10):5056–63. doi: 10.1021/acsenergylett.4c01727 (PMC11474943; doi:10.1021/acsenergylett.4c01727)
Supplement: Supplementary file 1 — nz4c01727_si_002.pdf [file nz4c01727_si_002.pdf]

Overcoming the Energy vs. Power Dilemma in Commercial Li-ion Batteries via Sparse Channel Engineering

*Doyoub Kim[a], Alexandre Magasinski[a], Yueyi Sun[b], Baolin Wang[b], Aashray Narla[a], Seung-Hun Lee[c], Hana Yoo[c], Samik Jhulki[a], Ah-Young Song[a], Jinho Hah[a], Ting Zhu[b], Alexander Alexeev[b], Gleb Yushin[a],**

a School of Materials Science and Engineering, Georgia Institute of Technology, Atlanta, Georgia, 30332, United States

b Woodruff School of Mechanical Engineering, Georgia Institute of Technology, Atlanta, Georgia, 30332, United States

c R&D Center, Samsung SDI, 130, Samsung-ro, Yeongtong-gu, Suwon-si, Gyeonggi-do 16678, Republic of Korea

* Corresponding Author: yushin@gatech.edu

Diameter (μm)	Spacing (μm)	% Density
15	450	99.97
15	100	99.32
30	450	99.87
30	700	99.94
30	900	99.97
30	250	99.56
30	100	97.28
50	100	92.45
50	250	98.79
50	450	99.63
50	700	99.85
50	900	99.91
100	900	99.63

Table 1. Electrode material losses for selected patterns, demonstrating that very small electrode losses may be realized with proper pattern engineering (0.1- 8 wt. %).

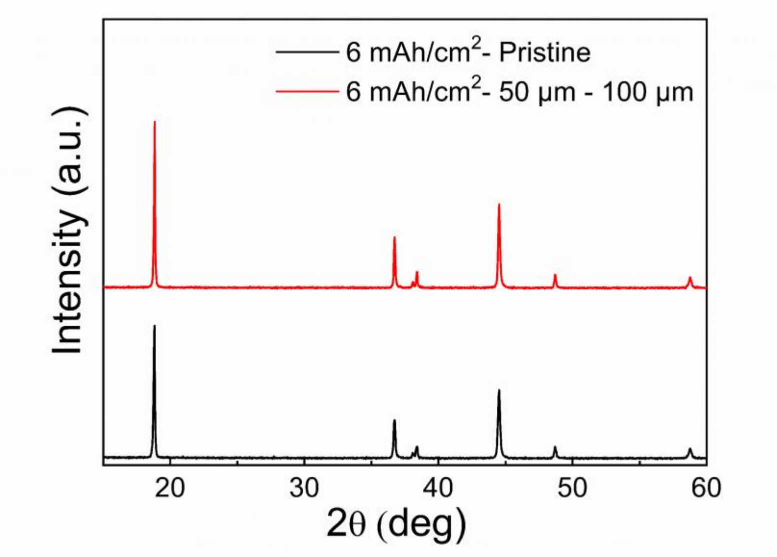

Figure S1. X-ray diffraction of the electrode before and after laser patterning shows no undesirable structural changes detected regardless of the residual laser heat.

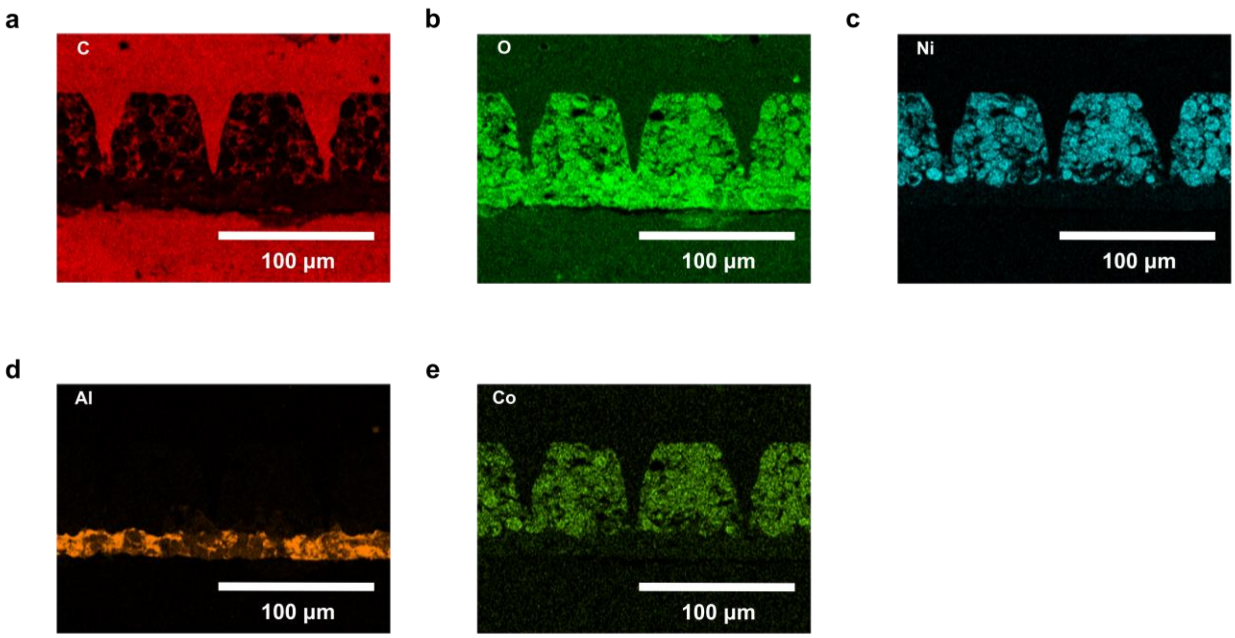

Figure S2. EDS of the cross-section of the laser-patterned electrode with conical-shaped channels shown in **Figure 1d**. Corresponding EDS elemental mapping of (a) C, (b) O, (c) Ni, (d) Al and (e) Co.

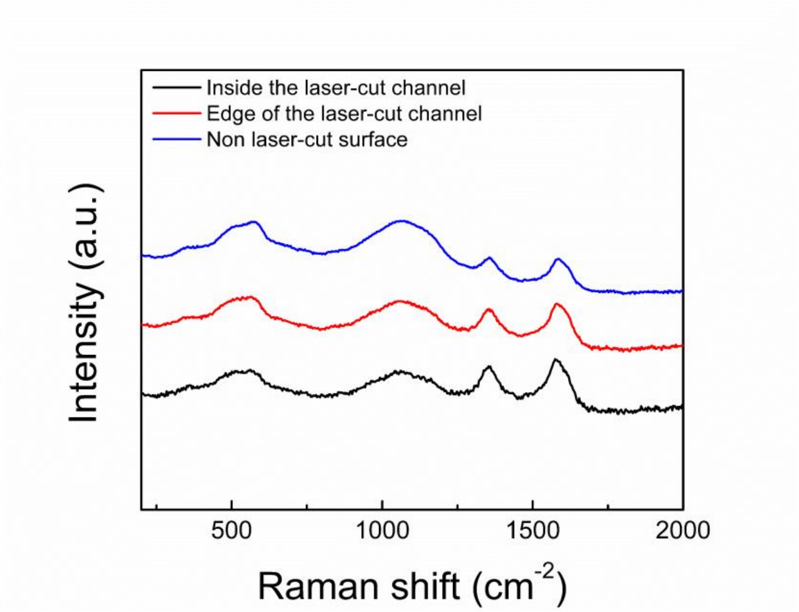

Figure S3. Raman spectra of specific areas of a laser-patterned electrode (50 μm channel diameter, 450 μm channel spacing).

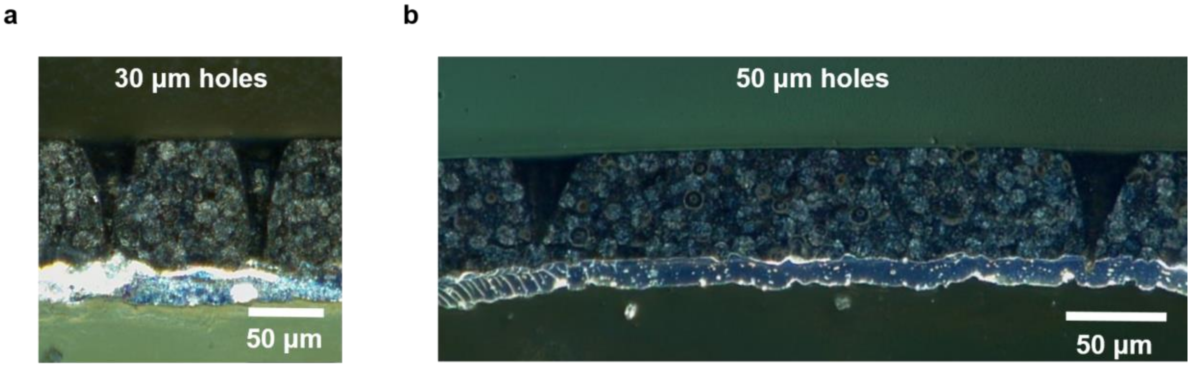

Figure S4. Optical images of the cross-section of laser-patterned 6 mAh/cm^2 electrodes with channel diameters of (a) 30 μm and (b) 50 μm with different spacings.

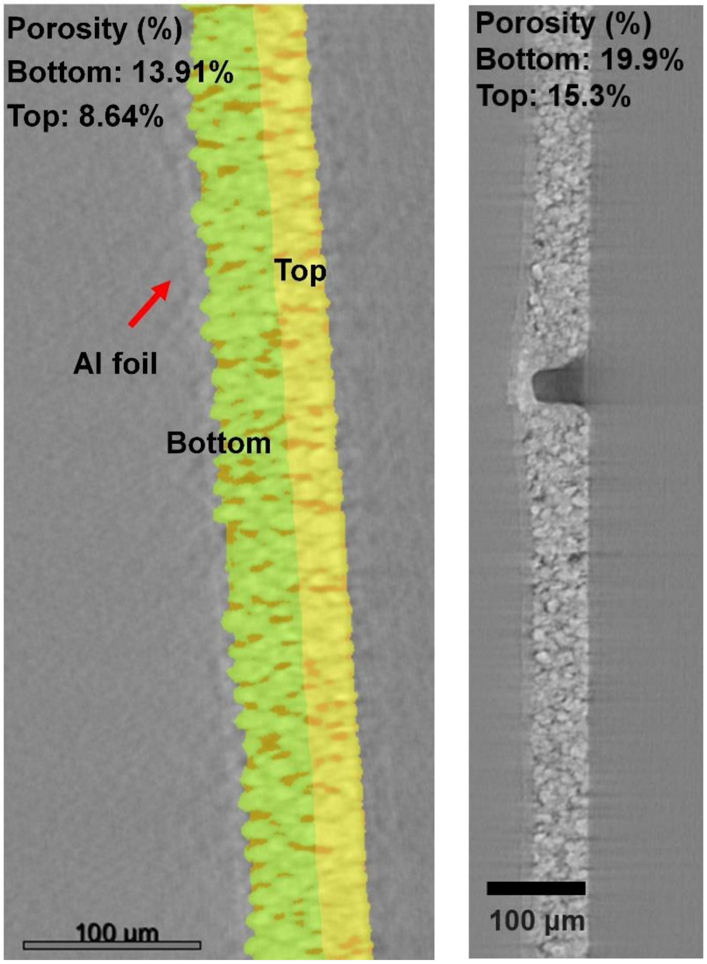

Figure S5. Nano-computed tomography (nano-CT) images of the cross-section of a (left) pristine and (right) laser-patterned (with 50 μm channel diameter) 4.8 mAh/cm^2 electrode and the measured porosity values for different layers of the electrode. The light green and yellow layers correspond to the bottom layer closer to the Al foil and the top layer further away from the Al foil, respectively.

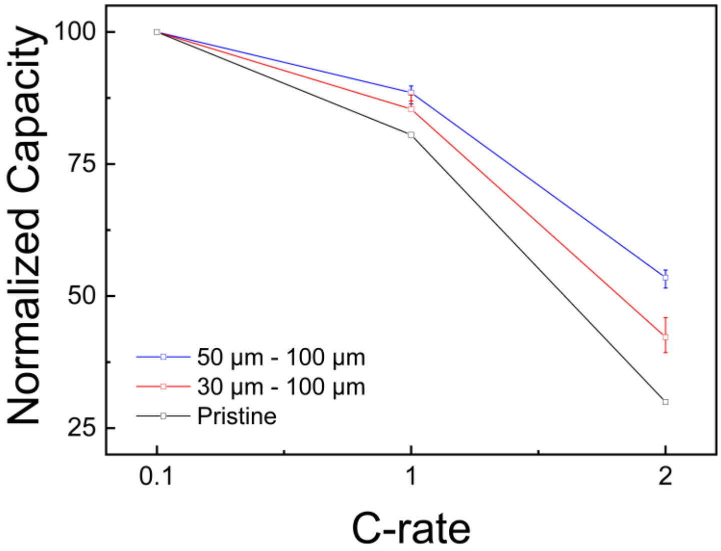

Figure S6. Figure 2d with error bars.

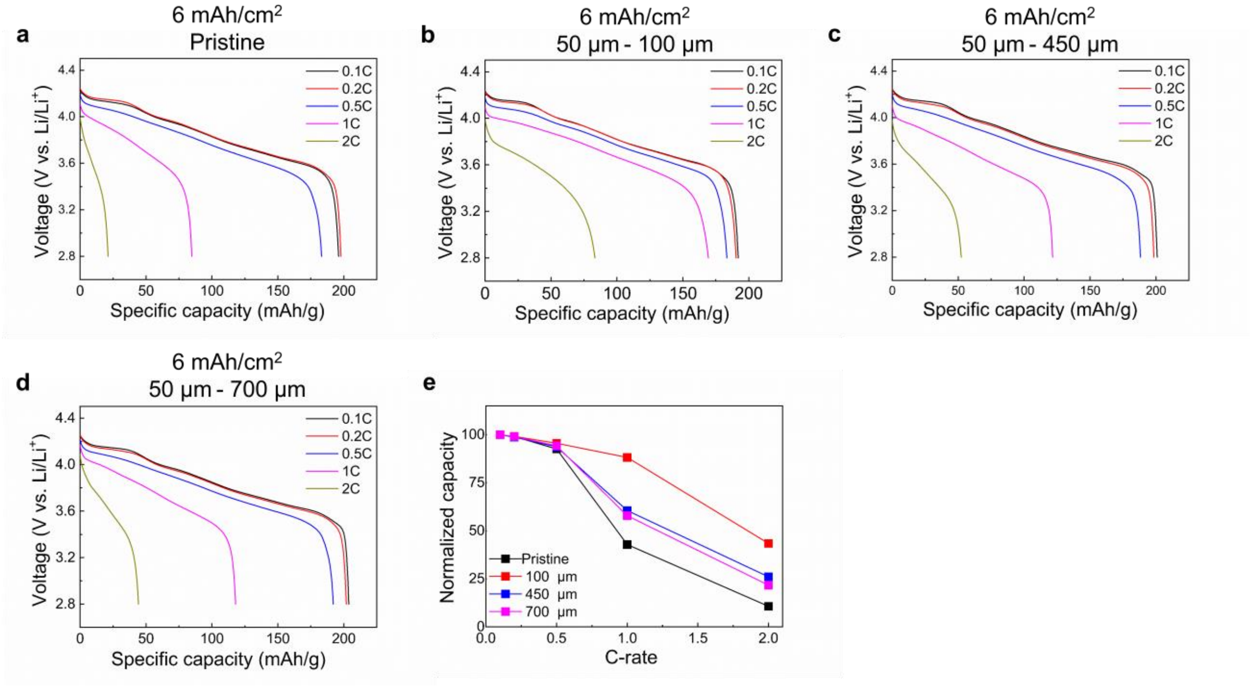

Figure S7. Electrochemical performance of pristine and laser-patterned 6 mAh/cm^2 electrodes. Discharge rate capability tests for (a) pristine, and laser-patterned (b) 50 µm – 100 µm, (c) 50 µm – 450 µm and (d) 50 µm –700 µm electrodes, where the charge rate was fixed at 0.1C cycle and 0.2C for discharges rates of 0.2C, 0.5C, 1C and 2C. (e) Discharge capacities obtained in (a), (b), (c) and (d) normalized by the capacities obtained at 0.1C.

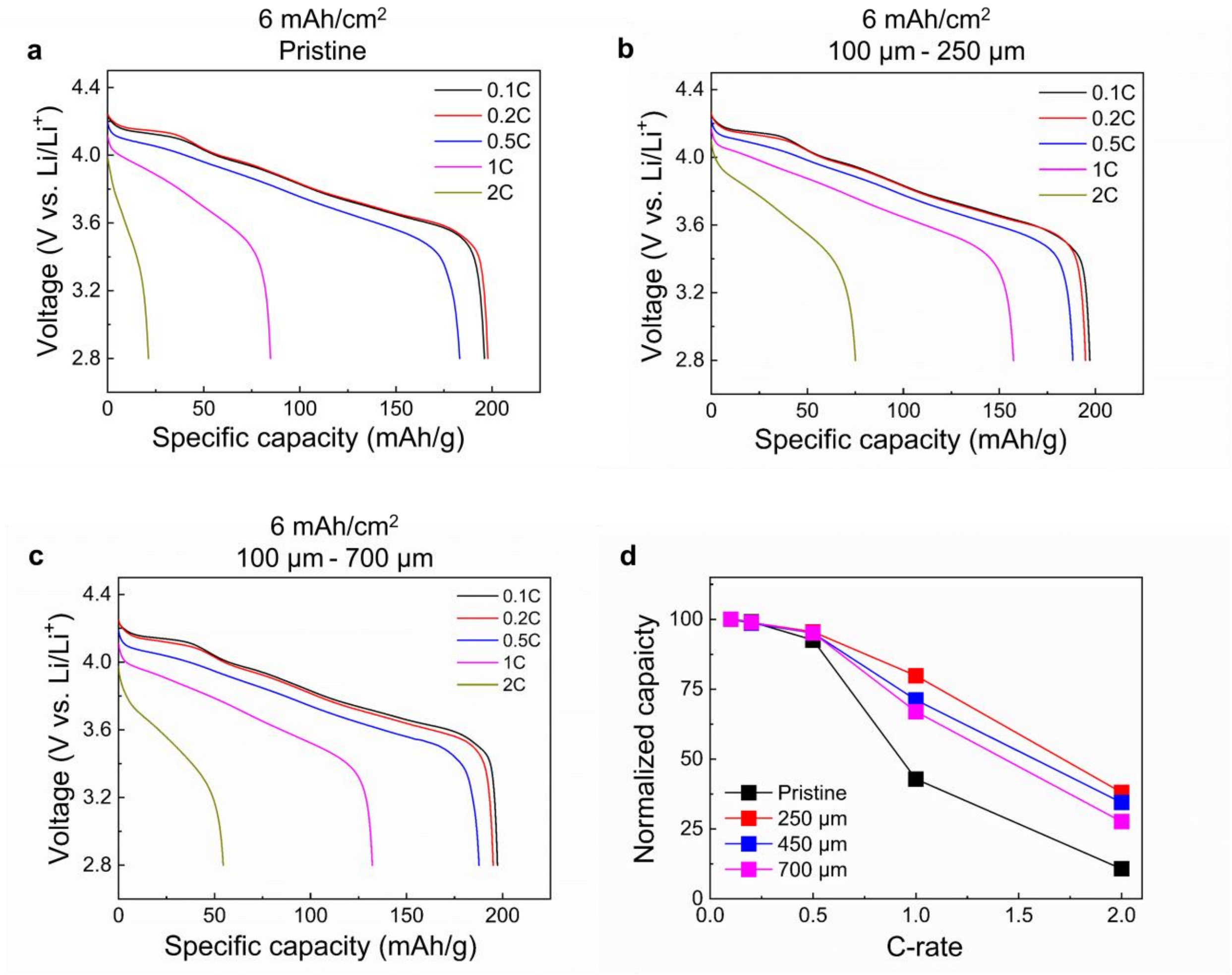

Figure S8. Electrochemical performance of pristine and laser-patterned 6 mAh/cm^2 electrodes. Discharge rate capability tests for (a) pristine, and laser-patterned (b) 100 μm – 250 μm and (c) 100 μm – 700 μm electrodes, where the charge rate was fixed at 0.1C cycle and 0.2C for discharges rates of 0.2C, 0.5C, 1C and 2C. (d) Discharge capacities obtained in (a), (b) and (c) normalized by the capacities obtained at 0.1C.

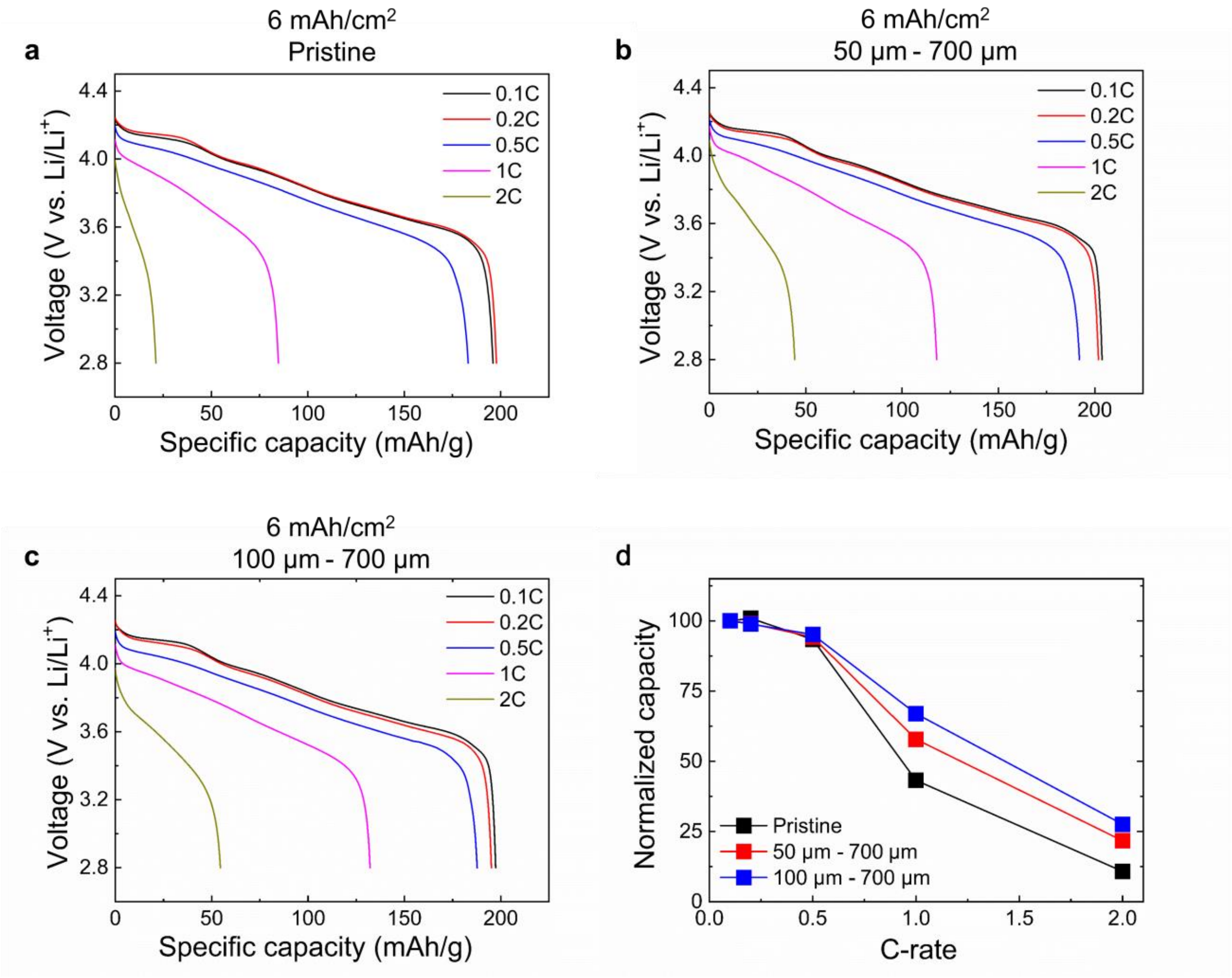

Figure S9. Electrochemical performance of pristine and laser-patterned 6 mAh/cm^2 electrodes. Discharge rate capability tests for (a) pristine, and laser-patterned (b) 50 µm – 700 µm and (c) 100 µm – 700 µm electrodes, where the charge rate was fixed at 0.1C cycle and 0.2C for discharges rates of 0.2C, 0.5C, 1C and 2C. (d) Discharge capacities in (a), (b) and (c) normalized by the capacities obtained at 0.1C.

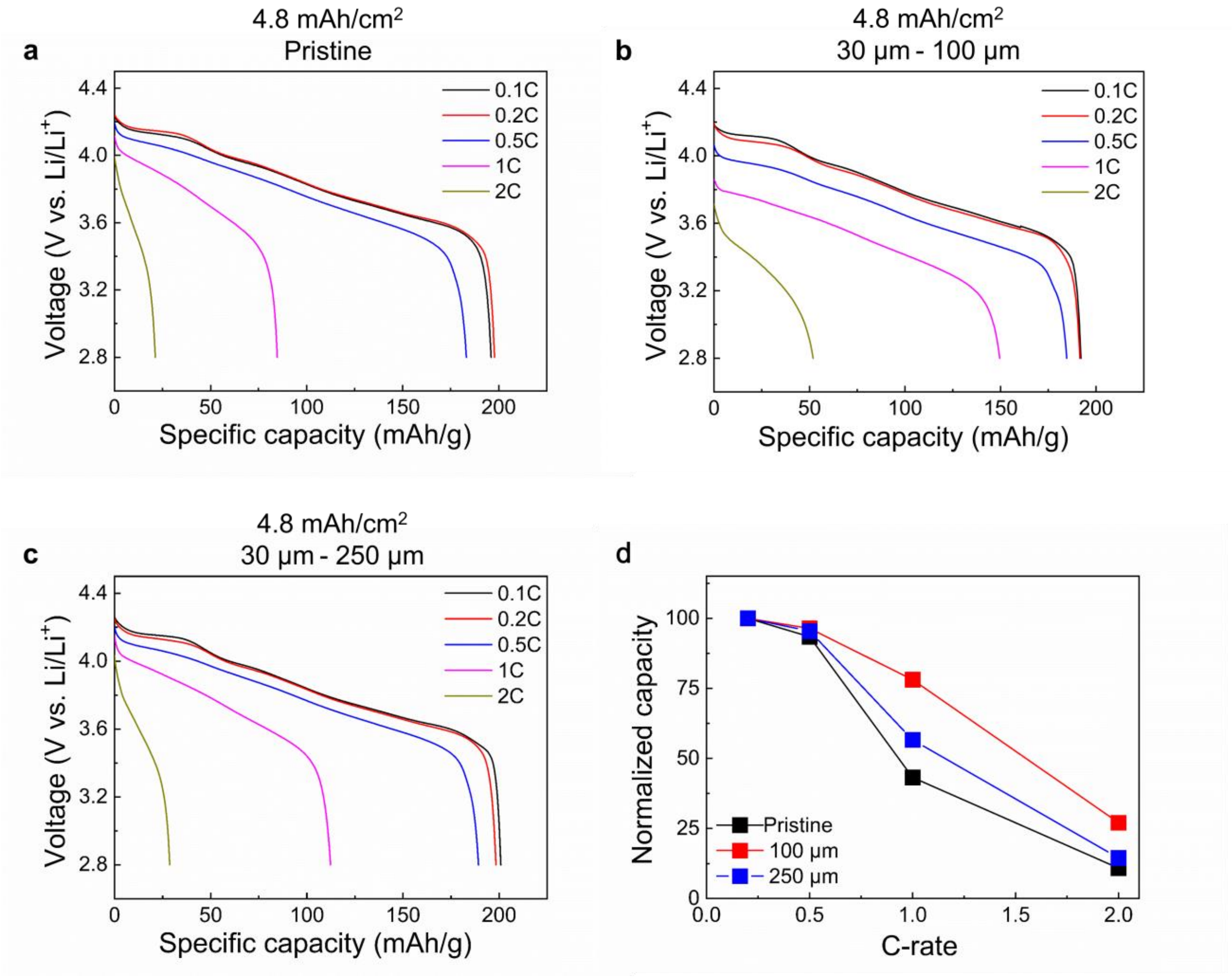

Figure S10. Electrochemical performance of pristine and laser-patterned 4.8 mAh/cm^2 electrodes. Discharge rate capability tests for (a) pristine, and laser-patterned (b) 30µm - 700µm (c) 30 µm – 250 µm electrodes, where the charge rate was fixed 0.2C for discharges rates of 0.2C, 0.5C, 1C and 2C. (d) Discharge capacities obtained in (a), (b) and (c) normalized by the capacities obtained at 0.2C.

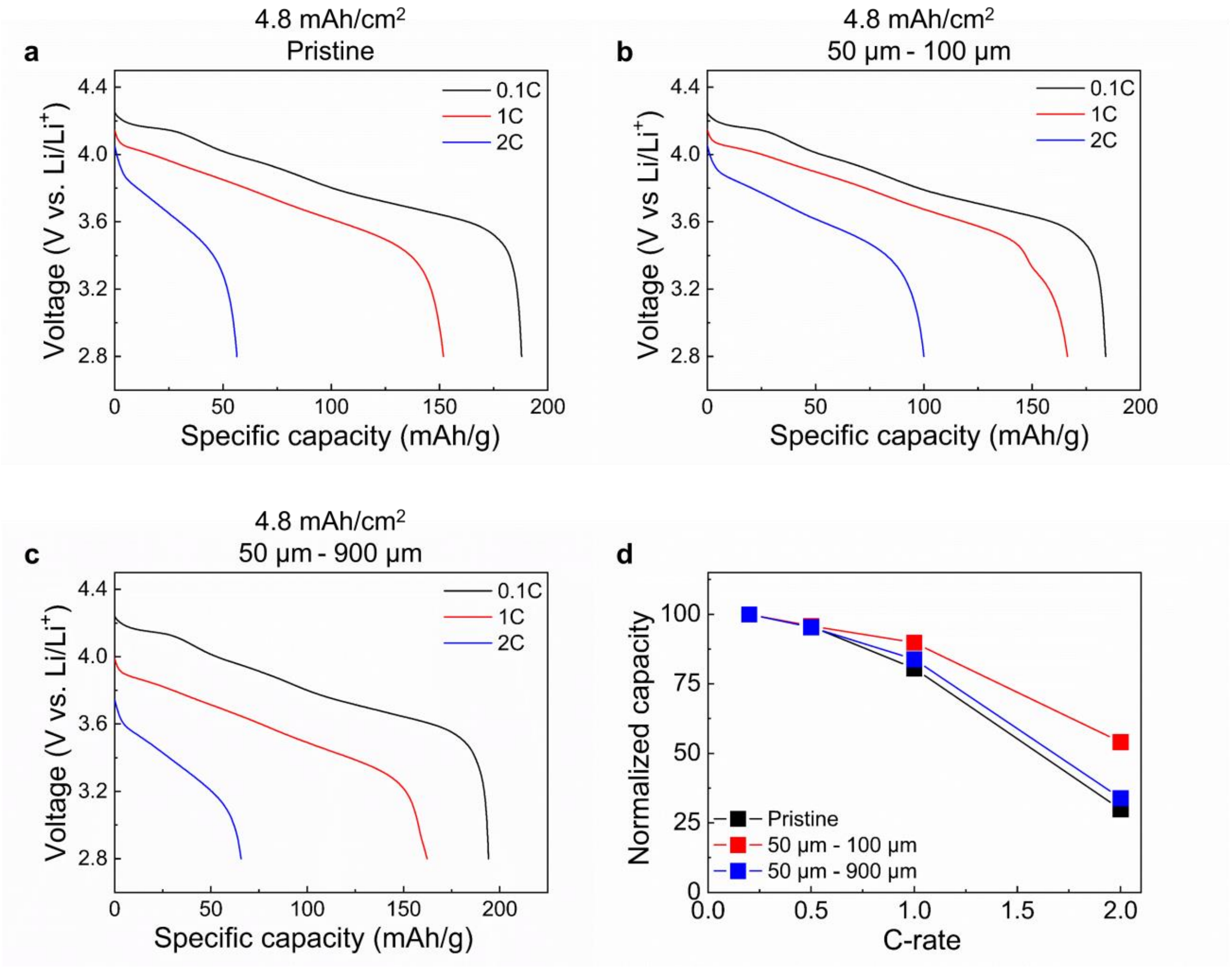

Figure S11. Electrochemical performance of pristine and laser-patterned 4.8 mAh/cm^2. Discharge rate capability tests for (a) pristine, and laser-patterned (b) 50 μm - 100μm (c) 50 μm – 900μm electrodes, where the charge rate was fixed at 0.2C for discharges rates of 0.2C, 0.5C, 1C and 2C. (d) Discharge capacities obtained in (a), (b) and (c) normalized by the capacities obtained at 0.2C.

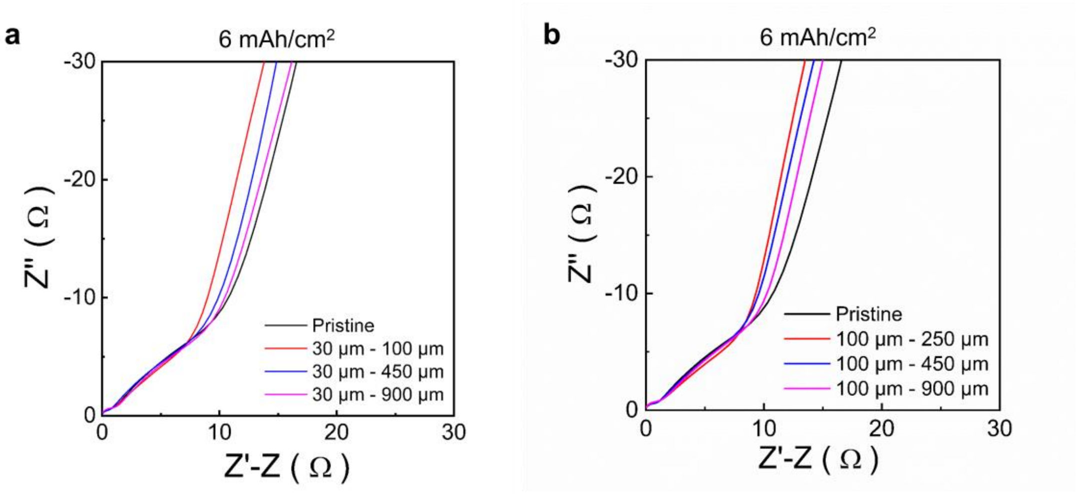

Figure S12. Effect of laser-patterning on tortuosity in relation to the channel size and the channel spacing on 6 mAh/cm^2 laser-patterned electrodes. The normalized Nyquist plots obtained for different laser-patterned channel spacings with fixed channel diameters of (a) 30 μm and (b) 100 μm.

4.8 mAh/cm^2

	Pristine	15	30	50	100
	24.99				
**100**		24.36	20.45	18.73	
**250**					20.95
**450**		24.58		22.68	21.38
**700**				23.68	21.75
**900**		24.98	24.54	24.13	24.01

Table 2. MacMullin numbers derived from linear fitting of impedance data from symmetric cells shown in Figure 4a and Figure 4b.

6 mAh/cm^2

	Pristine	15	30	50	100
	23.4				
**100**		19.31	15.25	14.29	
**250**				16.45	14.69
**450**		22.51	21.37		16.85
**900**		23.04	21.75	21.14	18.17

Table 3. MacMullin numbers derived from linear fitting of the impedance data from symmetric cells shown in Figure 4c and Figure 4d.

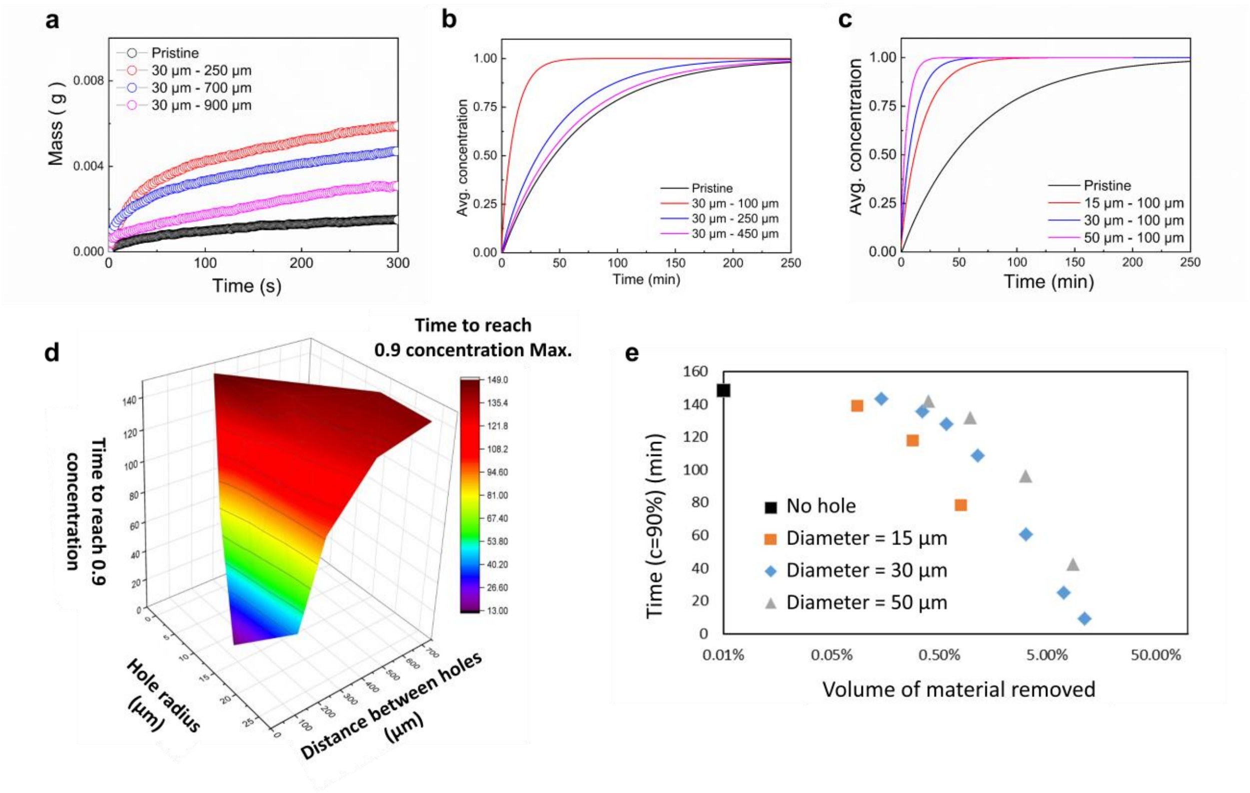

Figure S13. Effect of laser-patterning on electrolyte wetting and average lithium concentration. Immersion tests showing electrolyte impregnation in relation to laser-patterned electrodes as for variable (a) channel spacings in 1M $LiPF_6$ EC: DEC (V:V = 1:1). COMSOL simulation results of the pristine and laser-patterned electrodes with different channel spacings with a fixed channel diameter of (b) 30 µm and (c) 50 µm. Plots showing (d) time needed to reach average concentration of 90 % of maximum concentration in the lower layer of the electrode as a function of channel size and spacing and (e) volume percentage of the material removed by laser-patterning.

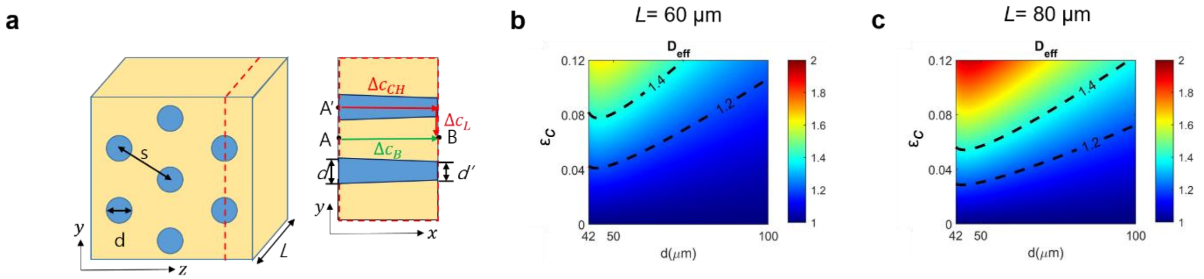

Figure S14. Effect of laser-patterning on electrodes with different thicknesses and uniform porosity. (a) Geometry of a hexagonal array laser-patterned electrode with a uniform total porosity of 14% for the entire electrode. Contour plots of normalized effective diffusivity based on steady-state electrolyte concentration drop across the hexagonal array laser-patterned electrode with thicknesses of (b) 60 µm and (c) 80 µm.

Experimental Methods

Preparation of electrode

This study is conducted with high Ni (> 80%) containing lithium nickel cobalt aluminum oxide (NCA) electrode supplied by Samsung SDI Co Ltd. Two types of electrodes were explored with areal capacities of 4.8 mAh/cm^2 and 6 mAh/cm^2, respectively, and both have the active material > 95 wt. %. WS-FLEX IR femtosecond laser is used with different variations of laser frequency, power speed and repetitions to optimize and obtain the most controllable hexagonal array pattern with minimal unwanted damage to the electrodes. The laser micro-machine is used to prepare AUTOCAD design to make hexagonal array patterns appropriately.

Material Characterizations

The Panalytical XPert PRO Alpha-1 XRD was used for all XRD experiments to examine the structural change and impurity formation before and after the laser micromachining. Data was collected for the 2θ range of 15–60°. Cross-section sample was first immersed in epoxy and left for up to one day to harden. The laser-micromachined NCA is individually mounted using two-part epoxy resin and hardener mixture in 1:0.23 (wt./wt.) ratio. These samples were ground using a series of different SiC papers up to 4000 grits and polished on PoliCloth (Buehler, USA) using 1 μm diamond suspension followed by polishing on Buehler VibroMet 2 using 0.05 μm colloidal silica suspension. During grinding and polishing steps, samples were thoroughly washed with acetone and rinsed with isopropanol (IPA) to remove residue and stains. The polished samples were then characterized using Leica digital microscope to obtain cross-sectioned images. Scanning electron microscopy (SEM) SU8010 was used to observe the channel size and spacing after laser micro-machining from the top using a standard SEM stub while SU8230 was used to examine the

cross-sections of the electrode to determine the shape of the channels and elemental distribution of the cross-sections using the EDS capability. 3D confocal microscopy was used to examine the pore shape of laser micromachined electrodes with different channel size and spacing variations. 3D pictures of electrodes and templates were captured with Olympus LEXT OLS 4000 laser confocal microscope (Olympus Scientific Solutions Americas Inc., USA) with a fixed laser wavelength of 405 nm and using magnification ranges: ×108 – ×17,000. The microscope was also used to measure the geometrical properties (measurement accuracy ca. ±1 mm) of the hexagonal array of channels. For each sample, three measurements were carried out at different positions of the electrode and the average data were used for all calculations. Immersion test was conducted to observe electrolyte impregnation before and after laser-patterned electrodes with different channel size and spacing. The experiments were conducted using pre-cut strips of 15 mm width using a Sigma 700/701 tensiometer (Biolin Scientific AB, Beijing, CPR), and a 1M $LiPF_6$ EC:DEC (V:V =1:1) electrolyte (Battery, Millipore Sigma, USA) was used as a wetting liquid. The measurement process is systematically controlled by a computer, and the mass of the electrolyte adsorbed by the electrode is recorded over time. A weight versus time (m-t) curve was constructed, from which the wettability of the electrode with the electrolyte is calculated. Porosity measurements were compared by collection X-ray tomography scans of the samples using a Zeiss Xradia 520 Versa. These scans were conducted at a higher resolution using a 4x objective lens, with a pixel size of 0.7 μm a power of 60/5 kV/W for a duration of 13 hours. The scans covered an area of approximately 1.4 × 1.4 mm. The process of thresholding was carried out to segment the pores and particles while eliminating minor noise.

Electrochemical Tests

After one cycle of C/10 and C/5 rates, followed by each cycle with varying discharge rates (C/2, 1C, 2C, 3C) is conducted while the charge rate is fixed at C/5. In between each charge and discharge cycle, there is a 10-minute gap allowed for equilibration. To study the impedance and tortuosity of the electrodes, electrochemical impedance spectroscopy was performed at an open circuit potential after a preliminary rest period of 24 h after making the fresh half cells. Impedance data were collected in a galvanostatic mode, and the frequency range was selected from 100 kHz to 10 mHz taking 10 points per decade. The Nyquist plots were measured with the 10 mV amplitude of the AC voltage. The electrochemical properties of the samples were assessed by employing a symmetric cell assembly to determine the MacMullin number. The symmetric cell assembly comprised a laser counter electrode measuring 5/8 inch, a working electrode measuring 1/2inch and Celgard 2400 with 1.0M $LiPF_6$ EC:DEC (1:1 = V:V, Sigma Aldrich) electrolyte. The tortuosity (τ) and the MacMullin number (MN) were determined using the resistivity of the electrolyte (1.32 Ω·m) and the observed resistivity of the samples. The relationship between tortuosity and MacMullin number is expressed as τ = MN × ε, where ε represents the porosity of the sample. The formula for calculating resistivity is $\frac{R_{ion}}{3}$ (Ω) = R-R_o, where R_{ion} is the resistance of the material with the electrolyte, and R_o is the resistance of the electrolyte. The resistivity (Ω·m) can be calculated using the formula ($\frac{R_{ion} \times A}{d}$, where A is the cross-sectional area of the material and d is the length of the material. MN is the ratio of the resistivity of the material to the resistivity of the electrolyte.

Simulation methods

We model Li-ion diffusion within a hexagonal electrode element of fixed height 80 μm and various side lengths $100 - 700$ μm using COMSOL. Periodic boundary conditions are applied in horizontal directions. We construct modeled electrode element with a less porous top layer (porosity 8%) and a more porous bottom layer (porosity 15%) to match the experimental observed electrode configurations (**Figure S5** in the Supporting Information). We include top-down holes with diameters $10 - 50$ μm on the four corners of modeled electrode element by setting porosity 100% for the holes. Transport of Li-ion within modeled electrode element is governed by $\partial(\epsilon_{p,i}c)/\partial t + \nabla \cdot [(-D_{Li+} \times \epsilon_{p,i}/\tau_{F,i})\nabla c] + u \cdot \nabla c = S$. Where c, $\epsilon_{p,i}$, $\tau_{F,i}$, D_{Li+} and S are the concentration of Li-ion, porosity and tortuosity of electrode layer material, Li-ion diffusion coefficient and source. Once we attach the top surface to a reservoir, Li-ions diffuse into modeled electrode element. We analyzed the effect of hole sizes and patterns on Li-ion concentration and total Li-ion count within modeled electrode element over time.

A model using a finite element method was developed to consider the porous electrode structure with a periodic array of conical channels as shown in **Figure 1d**. The electrode thickness L and the porosity of the porous electrode matrix (yellow part in **Figure S5**) ε_b are fixed since they are (almost) unchanged before and after the laser micromachining. The remaining parameters include the top-hole diameter d, spacing s and bottom-hole diameter d′ determine the energy density and areal capacity of the electrode. Based on Fick’s first law of diffusion, $J = D_{eff}\nabla C$, we know that when the flux J is fixed, the effective diffusivity D_{eff} is inversely proportional to the steady state concentration drop across the electrode ΔC. Therefore, the rate of ion transport indicated by D_{eff} can be evaluated by computing ΔC. The steady state concentration drop across the electrode is

calculated from the modified porous electrode model.[1,2] In our calculations, the classic Bruggeman relationship between tortuosity τ and porosity ε, $\tau = \varepsilon^{-0.5}$, is employed. From the cross-section view of the electrode in Figure 4e, there are two limiting paths for ion transport from point A (next to separator) to point B (next to current collector). One path (red arrow) is through the porous electrode matrix alone and parallel to the channel; the other one (green arrow) is first along the channel and then laterally across the porous electrode matrix. An effective concentration drop ΔC_{eff} is computed by the addition rule of parallel resistors from ΔC through the two paths. The contours for the effective diffusivity D_{eff} (inverse of ΔC_{eff}) is shown in **Figure 4f** and **S14** as a function of the porosity of the channels ε_c and hole-diameter d for ε_b=0.14 and L=80 μm. The effective diffusivity D_{eff} is normalized by that before laser treatment (electrode without channels) such that this value indicates the increase of D_{eff} after laser treatment. The porosity of the channels ε_c represents a measure of loss of active materials due to laser treatment. It is related to s by equation $\varepsilon_c=(d^2+d'^2+d\cdot d')/3s^2$ for a specific set of d and d' by which the shape of the conical channels is determined. In the computation of Fig. 4g, the slope of the surface is assumed to be fixed which means the difference between d and d' is constant.

References

(1) Doyle, M.; Newman, J. Analysis of capacity–rate data for lithium batteries using simplified models of the discharge process. *Journal of Applied Electrochemistry* **1997**, *27* (7), 846-856. DOI: 10.1023/A:1018481030499.

(2) Bae, C.-J.; Erdonmez, C. K.; Halloran, J. W.; Chiang, Y.-M. Design of Battery Electrodes with Dual-Scale Porosity to Minimize Tortuosity and Maximize Performance. *Advanced Materials* **2013**, *25* (9), 1254-1258. DOI: https://doi.org/10.1002/adma.201204055.
